# Developing a Smart Sensing Sock to Prevent Diabetic Foot Ulcers: Qualitative Focus Group and Interview Study

**DOI:** 10.2196/59608

**Published:** 2025-02-14

**Authors:** Jenny Corser, Irantzu Yoldi, Neil D Reeves, Pete Culmer, Prabhuraj D Venkatraman, Giorgio Orlando, Rory Peter Turnbull, Paul Boakes, Eric Woodin, Roger Lightup, Graham Ponton, Katherine Bradbury

**Affiliations:** 1 Centre for Clinical and Community Applications of Health Psychology University of Southampton Southampton United Kingdom; 2 School of Health, Sport & Bioscience University of East London London United Kingdom; 3 Medical School Faculty of Health and Medicine Lancaster University Lancaster United Kingdom; 4 School of Mechanical Engineering University of Leeds Leeds United Kingdom; 5 Manchester Fashion Institute Faculty of Arts and Humanities Manchester Metropolitan University Manchester United Kingdom; 6 Department of Sport and Exercise Sciences Institute of Sport, Faculty of Science and Engineering Manchester Metropolitan University Manchester United Kingdom; 7 SOCKSESS patient advisory group University of Southampton Southampton United Kingdom; 8 NIHR ARC Wessex National Institute for Health Research London United Kingdom

**Keywords:** diabetes, diabetic neuropathy, diabetic foot ulcer, podiatry, prevention, health technology, behavior change

## Abstract

**Background:**

Diabetic foot ulcers are common and costly. Most cases are preventable, although few interventions exist to reliably support patients in performing self-care. Emerging technologies are showing promise in this domain, although patient and health care provider perspectives are rarely incorporated into digital intervention designs.

**Objective:**

This study explored patient and health care provider feedback on a smart sensing sock to detect shear strain and alert the wearer to change their behavior (ie, pause activity and check their feet) and considered how patient experience and attitudes toward self-care are likely to impact uptake and long-term effective engagement with the device to curate guiding principles for successful future intervention development.

**Methods:**

This qualitative study combined semistructured interviews and a focus group alongside a participant advisory group that was consulted throughout the study. In total, 20 people with diabetic neuropathy (n=16, 80% with history of diabetic foot ulcers) and 2 carers were recruited directly from podiatry clinics as well as via a recruitment network and national health mobile app for one-to-one interviews either in person or via landline or video call. A total of 6 podiatrists were recruited via professional networks for 1 virtual focus group. Participants were asked about their experience of diabetic foot health and for feedback on the proposed device, including how it might work for them in daily life or clinical practice. The data were analyzed thematically.

**Results:**

Three main themes were generated, each raising a barrier to the use of the sock complemented by potential solutions: (1) patient buy-in—challenged by lack of awareness of risk and potentially addressed through using the device to collect and record evidence to enhance clinical messaging; (2) effective engagement—challenged by difficulties accepting and actioning information and requiring simple, specific, and supportive instructions in line with podiatrist advice; and (3) sustained use—challenged by difficulties coping, with the possibility to gain control through an early warning system.

**Conclusions:**

While both patients and podiatrists were interested in the concept, it would need to be packaged as part of a wider health intervention to overcome barriers to uptake and longer-term effective engagement. This study recommends specific considerations for the framing of feedback messages and instructions as well as provision of support for health care providers to integrate the use of such smart devices into practice. The guiding principles generated by this study can orient future research and development of smart sensing devices for diabetic foot care to help optimize patient engagement and improve health outcomes.

## Introduction

### Background

Foot ulceration is a common and debilitating problem for people with diabetes and is costly to the health care system. Up to one-third of individuals with diabetes will develop a foot ulcer in their lifetime [1], and amputation or death is likely in up to half of those individuals within 5 years [2]. These adverse outcomes understandably impact patient mental health, and it is reported that one-third of people experience clinical depression with their first diabetic foot ulcer [3]. In the United Kingdom, for the year 2014 to 2015, diabetic foot disease cost the National Health Service (NHS) 1% of its entire budget [4]. Indirect costs include impacts on individual earnings, costs of carers, and absenteeism for employers [5]. Despite many ulcers being preventable [6], only a fraction of health care spending is on prevention [7,8]. It is estimated that preventing one-third of ulcers in England would save the UK NHS >£250 million (US $325 million) [4].

Digital interventions show promise for supporting foot ulcer prevention. Emerging technologies include wearable devices such as smart insoles or smart socks that can be worn daily to provide constant monitoring of the feet and alert the wearer to at-risk foot loading [9-12]. Tests of these technologies show that regular use could be effective in predicting ulceration [9] and that participants find smart socks comfortable, yielding a good compliance rate [13,14]. Socks may be preferable to insoles as they can be worn with any type of footwear (or indeed on their own) [15]. Current smart wearable devices (socks and insoles) monitor temperature and plantar pressure, but research suggests that results would be improved by measuring shear strain, which reflects the “rubbing” across the foot [16,17]. Technology that measures shear strain has only been developed bespoke for research purposes, and application to wearables in this population is currently unavailable [18,19]. Recently, insoles capable of measuring shear safely have been developed and laboratory tested [20-22], but no studies have yet been found to measure shear strain via socks.

### Objectives

A recent systematic review of smart wearable technology in diabetic foot ulcer prevention highlighted the limited involvement of patient and health care provider perspectives in device design and evaluation [23]. It is not surprising, then, that there is a lack and urgent need of interventions addressing patient barriers to adherence [24], and this requires patients and health care providers involved in diabetic foot health care to be consulted throughout the design process [25]. If the aim is to support effective engagement with a device [26] and improve health outcomes, interventions should carefully consider not only usability of features but whether the technologies are likely to change critical behaviors [27]. For example, it is important that users are supported not only in wearing the device but also in responding to it appropriately (ie, offloading the foot or seeking medical help if an ulcer has developed). This study used qualitative data to facilitate the co-design of a novel solution for daily monitoring and prevention of diabetic foot ulcers (a smart sock to detect shear strain and an associated feedback system). The aim of this study was to better understand the needs and preferences of those who would use or support the use of the technology to inform decisions about what would be needed to make a shear-sensing smart sock most likely to be adopted and adhered to in the long term and maximize the potential patient benefit. This included exploring lived experiences of diabetic foot ulcers as well as direct feedback on the proposed technology. This paper summarizes our findings thematically and includes a related set of guiding principles for future research and practice in smart sensing devices for diabetic foot care.

## Methods

### Study Design

Qualitative data were collected via semistructured interviews and a focus group in parallel to the technology development and used to iteratively inform its progress. In addition to participant input, regular patient and public inclusion and engagement (PPIE) opportunities with a patient advisory group of 8 people living with diabetes and presenting with diversity in severity of diabetic neuropathy (and consequent risk of diabetic foot ulcers) were held at regular intervals throughout the study period.

The role of the PPIE group was to provide lived experience input and early advice to the research team to help shape the study in the early phases (eg, co-designing and piloting the interview schedule) and throughout the data collection and analysis phases for credibility checking and feedback. Finally, they reviewed and provided input on the authorship of this publication. Members were recruited via professional networks and snowballing during the grant and ethics application phases of the study. The group met 5 times over 12 months.

### Ethical Considerations

Ethics approval for this study was obtained from the University of Southampton (Ethics and Research Governance Online 78959), the UK Health Research Authority (Integrated Research Application System 323631), and the local research ethics committee (South Central – Hampshire B Ethics Committee; 23/SC/0098). The procedures followed were in accordance with the ethical standards of the responsible committee on human experimentation and with the Helsinki Declaration of 1975 as revised in 2000. All participants took part after completing an informed consent procedure, with the possibility to opt out of the study at any time. All references to participants and their data have been anonymized to protect their privacy. The participation of the PPIE group was voluntary, with no contractual obligations, and they were paid £25 (US $31.25) per hour of involvement. Participants were offered a £25 (US $31.25) gift voucher as a thank you.

### Participants

Potential users of the technology were identified to be people with diabetes and neuropathy and, therefore, at risk of developing diabetic foot ulcers who might use the sock and feedback system on a daily basis; their carers who might facilitate this daily use; and podiatrists (although various health care providers may be involved in diabetic foot care, podiatrists are most likely to implement the technology in clinical practice and have the most specialized knowledge in the area for device feedback). Recruitment began in May 2023 (month 7 of the study) and was completed in December 2023 (month 13 of the study).

#### Patients and Carers (for Interviews)

People with diabetes were recruited via postal mail-out from NHS podiatry clinics. Although the invitations were targeted to patients, carers were also invited to participate. Invitation packages included a cover letter with a brief summary of the study and contact information and a full participant information sheet detailing potential risks and data governance. Patient participants were included if they had diabetes and reported changes in sensation in their feet. Interested participants contacted the research team directly to ask questions, find out more about the study, and provide contact details for participation.

In addition to invitations from the clinic, the study was also posted on the NHS app, and an additional recruitment stream was set up using a consent-for-approach recruitment service (National Institute for Health and Care Research Clinical Research Network, Research for the Future).

With an aim to understand barriers to equitable engagement with the technology and mitigate them through its design, participants were selected purposively to include a range of ages, gender identities, ethnicities, and relative deprivation levels (based on the Index of Multiple Deprivation score [28] from their address), with an aim to oversample from underserved groups (eg, groups of a lower socioeconomic status and non-White ethnicity).

Those who were eligible were invited to be interviewed either in person in their homes or remotely via teleconferencing software or via landline telephone. On the basis of previous similar projects, a sample size of 20 to 30 patients and carers was estimated to provide sufficient information power [29]. Diversity of perspectives, depth of insight through strong dialogue, and rich data collection were prioritized over achieving a specific sample size.

#### Podiatry Group (for Focus Group)

Podiatrists working with people with diabetes were recruited via professional networks. Information about the study was made available via the clinics that were recruiting patients and via emails to colleagues. Interested participants contacted the research team directly to ask questions, express interest, and indicate availability to participate.

### Data Collection

One-to-one interviews were conducted by JC (a qualitative researcher and lead author) in person in the participants’ homes (6/22, 27%) or via teleconferencing (11/22, 50%) or phone (5/22, 23%) where preferred. Each participant was interviewed once. Before recording, the researcher reviewed the purpose of the study. Participants were given the opportunity to ask questions and then asked to complete the consent form followed by a demographic questionnaire including questions about their age, gender identity, living arrangements, and medical history. Participants were advised that specific questions about the technology were asked in terms of co-design, as if they were designing it for their own personal needs, and there were no right or wrong answers. “Shear strain” was described as “rubbing,” and the researcher demonstrated this concept by rubbing the back of her hand and showing how the skin “stretches.”

A semistructured interview guide with main questions and prompts was used and initially piloted and refined with the PPIE group (Multimedia Appendix 1). The interviews began by asking about the participants’ experience with their foot care—previous issues, how they managed their foot care, and what they understood about diabetic foot health. The researcher then provided a standardized lay summary of the concept of the sock and feedback system (also developed with the PPIE group) with sock samples where available. The participants were encouraged to ask questions freely during and after the description. Participants were asked about their first impressions, whether the technology might fit into their daily life, how they would respond to alerts, and whether there were any concerns they had about the design or elements they would like to change. The interviews lasted an average of 52.5 (SD 11.0) minutes and were audio recorded and transcribed verbatim.

One focus group with podiatrists was conducted at month 12 of the study via the Microsoft Teams (Microsoft Corp) teleconferencing platform and facilitated by JC. Participants were sent 4 different sock samples and 1 sample of sensor material in the post before the discussion. The discussion began with a review of socks currently marketed for patients with diabetes and what the participants thought were important features for a sock designed for patients at high risk of diabetic foot ulcers. The concept of the sock and feedback system was presented orally using visual presentation slides. Participants were encouraged to speak freely about their first impressions of the technology in general, specific features, and implications for practice. The focus group lasted 70 minutes and was audio recorded and transcribed verbatim. Field notes and a reflective diary were kept throughout the data collection period.

### Data Analysis

**Figure 1:**
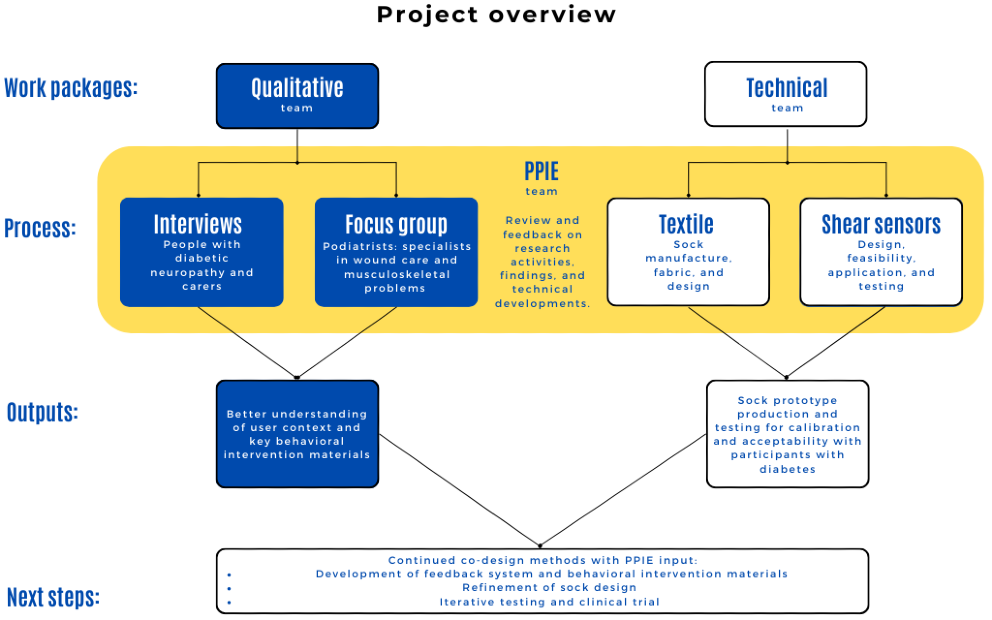
Division of work streams within the Socksess project and their interactions. PPIE: patient and public inclusion and engagement.

Data were collected over 5 months and were initially coded by the main author as positive and negative comments about the socks. These comments were presented to the PPIE group and the wider research team, including engineers of the sensors and manufacturers of the socks, for feedback. A brief summary of these findings is presented in Multimedia Appendix 2, and Figure 1 illustrates the parallel nature of this qualitative data collection and central role of PPIE input alongside the technical development of the sock by the wider research team. This ongoing process allowed for new data to be compared with previously collected data to identify similarities and deviances that were relevant and helpful to consider in the technology development process. Once all data had been collected, an overview and in-depth reflexive thematic analysis was conducted by JC guided by the principles of Braun and Clarke [30].

As JC collected and transcribed the data and had reviewed each case for feedback and discussion with the PPIE group, she was already familiar with the data by the stage of full analysis when attentional focus turned to the transcripts and field notes as a corpus. Codes were generated inductively using the NVivo software (QSR International) [31]. As the podiatrist data were more technical than the interview data and focused more on elements of the technology rather than on patient context, these data were assessed in parallel as a unique perspective separate from but related to the patient perspective. Throughout the coding process, the researcher made reflective notes.

Once generated, the codes and researcher notes were assessed together as a corpus. Throughout the process of data collection, JC learned about the experience of diabetic foot ulcers and developed empathy for the participants regarding the challenges of peripheral neuropathy and self-management of ulcer treatment and prevention. JC drew on the personal impact of these stories while analyzing the data to generate themes describing salient aspects of the experience of diabetic foot disease and how a novel technology such as this one may work in the everyday lives of people managing it. Initial themes were drafted and presented to the PPIE group and the larger research team for discussion and were reviewed and refined iteratively. PPIE engagement was essential to this refinement process, developing the themes in a way that presented a credible and relevant narrative.

To ensure the quality of data reporting, the COREQ (Consolidated Criteria for Reporting Qualitative Research) guidelines were followed [32]. A copy of the checklist, including a reflexivity statement, can be found in Multimedia Appendix 3.

## Results

### Recruitment

A total of 22 participants were recruited for the interviews, including 20 (91%) participants with diabetic peripheral neuropathy (n=13, 59% identified as male; n=8, 36% identified as female; and n=1, 5% identified as transgender), of whom 5 (23%) had type 1 diabetes and 17 (77%) had type 2 diabetes. Participants had a mean age of 66.0 (SD 10.5) years and a mean diabetes duration of 21.6 (SD 12.1) years. Of these participants, 73% (16/22) had a previous history of ulceration, 27% (6/22) had a previous history of amputation, and 14% (3/22) had a diagnosis of Charcot neuroarthropathy. Participant characteristics are summarized in Table 1.

**Table 1:** Interview participants (N=22)^a^.

Characteristic		Values
**Participant type, n (%)**		
	Patient	20 (91)
	Carer	2 (9)
**Gender identity, n (%)**		
	Man	13 (59)
	Woman	8 (36)
	Transgender	1 (5)
**Patient age (years; n=20), n (%)**		
	36-45	1 (5)
	46-55	3 (15)
	56-65	2 (10)
	66-75	8 (40)
	76-85	6 (30)
**Ethnicity, n (%)**		
	Asian (Indian, Pakistani, Bangladeshi, Chinese, or any other Asian background)	3 (14)
	Black, African, or Caribbean	2 (9)
	Mixed (2 or more ethnic groups)	1 (5)
	White British	16 (73)
**IMD^b^ score, n (%)**		
	1	3 (14)
	2	2 (9)
	3	5 (23)
	4	2 (9)
	5	1 (5)
	6	1 (5)
	7	2 (9)
	8	0 (0)
	9	2 (9)
	10	4 (18)
**Housing, n (%)**		
	Living alone	9 (41)
	Living with at least one other family member	13 (59)
**Diabetes**		
	Type 1, n (%)	5 (23)
	Type 2, n (%)	17 (77)
	Duration (years), mean (SD)	21.6 (12.1)
**Years since diabetes diagnosis (n=20), n (%)**		
	1-10	3 (15)
	11-20	4 (20)
	21-30	6 (30)
	31-40	7 (35)
**Years since neuropathy diagnosis (n=20), n (%)**		
	1-10	11 (55)
	11-20	4 (20)
	21-30	3 (15)
	Not sure	2 (10)
**DFU^c^, n (%)**		
	Previous ulcers	16 (73)
	Amputation	6 (27)
	Charcot neuroarthropathy	3 (14)
**Perceived risk versus actual risk^d^, n (%)**		
	Underestimation	7 (32)
	Accurate estimation	9 (41)
	Overestimation	3 (14)

^a^The demographics listed include those of the patients and carers except for the health-related data, which are only provided for patients.

^b^IMD: Index of Multiple Deprivation score—a relative measure of deprivation for a small geographic area (single postcode) in the United Kingdom. Scores range between 1 (most deprived) and 10 (least deprived).

^c^DFU: diabetic foot ulcer.

^d^Participants were asked whether they thought their risk of another ulcer was low, medium, or high, and this was compared with the risk levels on the National Institute for Health and Care Excellence guidelines informed by their self-reported presence of neuropathy and history of ulcers. Self-report of symptoms usually exceeds diagnosis, and participants were often unsure or in denial. Responses were vague. Where a range was given, an average was used; where the response was “at least x years,” x was used.

A total of 6 Health and Care Professions Council–registered podiatrists were recruited. All currently worked in England (5/6, 83%) or Scotland (1/6, 17%), in the NHS (5/6, 83%), and academia (1/6, 17%). Participants had previous experience working in public and private health care systems as well as working overseas. Participants specialized in wound care (5/6, 83%) and musculoskeletal problems (1/6, 17%).

### Thematic Analysis Findings

#### Overview

This section presents a thematic analysis of participant feedback on the design concept of this device. In total, 3 themes were developed: patient buy-in, effective engagement, and sustained use. Each theme is split into 2 subthemes, the first highlighting a contextual challenge and the second presenting participant preferences for the intervention related to that challenge.

On presentation of the design concept, many participants appeared surprised that such a technology might exist, with comments such as “it would be a revolution, if it could work” (P17). The subsequent disbelief yielded questions and doubts about the sensitivity of the device:

...you know, a beep every five minutes you’re just gonna get plain fed up with it aren’t you? And then if you don’t find anything, you know your faith in the product is just going to diminish.

P16

This concern was understandably a pivotal factor for acceptability. As such, participants were asked to imagine using a device that was perfectly calibrated to them. The remainder of this section describes the themes in detail with quotations from participants.

#### Patient Buy-In

##### Lack of Awareness of Risk

Although most participants considered the idea of the sock to be interesting, participants who judged themselves to be at lower risk of ulceration or doubted that rubbing was a cause of foot injury for them needed more persuading:

Would I say I would go out and buy a pair of those socks? Not necessarily, because I don’t think I need to.

P8

The device is designed to target loss of sensation caused by diabetic neuropathy, and yet this was a particularly challenging symptom for participants to make sense of and describe. In cases in which participants believed that they had sensation in their feet, the diagnosis of neuropathy could be more challenging to accept cognitively, whereas the association with loss or inadequacy could also be difficult to accept emotionally:

You lose sensitivity in your feet to different degrees, I mean as far as I’m concerned, I fail the medical test where they put a hair across your feet to designate if there’s any feeling there, so I fail that, and I failed it for a long time, however in terms of if I stood on something, or if can I feel the pedals in the car, yes, I can.

P8

The podiatrist group also noted challenges with limited patient awareness and acceptance of risk—“they’re in denial about a lot of things” (podiatrist 3)—and consequent issues engaging these patients to actively participate in their foot health management:

...it’s a cohort of patients who don’t even do the basic kind of self-care stuff.

Podiatrist 1

Despite efforts to educate their patients in the clinic, they were aware that many of their patients struggled to follow the self-care instructions at home:

Essentially we’re there to help them heal, but at the end of the day their foot is at the end of their leg and that goes home with them. And what happens in between appointments is obviously based on what they do.

Podiatrist 4

##### Ability to Collect and Record Evidence

Without the ability to physically perceive shear strain occurring, people with neuropathy would not normally have the information to understand and detect how, when, or why damage occurred. This created confusion and doubt in some participants, who were unsure of how to make sense of their ulcers. Participants from both groups (interviews and focus group) thought that the sock could help elucidate issues regarding shear strain, thus clarifying misconceptions and reinforcing clinical messaging. The following quote is one participant’s response to being asked why their ulcers may have occurred:

I haven’t got a clue. I feel that there hasn’t been a common reason I’ve had these ulcers...There’s no plausible reason for why it’s happened. Anything that investigates that would be nice to know the results.

P19

Podiatrists thought that the sock could be useful in creating awareness and collecting information surrounding the time of alerts that would otherwise not be possible to obtain. Importantly, they felt that becoming aware of when the shear strain occurred might help patients (and clinicians) identify factors that could be controlled (eg, if it only happens at work when wearing steel-toe boots) and, ultimately, help the patient mitigate these risks themselves:

I would be thinking straight away what activity are they doing? Are they stationary? Are they, you know, walking along somewhere? Are they pottering around indoors? Because when is it rubbing? That’s because that’s the type of thing that I would ask in clinic, you know, with footwear. What were you doing?

Podiatrist 6

Lack of sensation limits not only the ability of patients to know what is happening with their feet in real time but also how they can communicate issues to others. Consequently, information that patients report in the clinic or at home is often not complete or reliable for the podiatrists or the carer to know how and when to proceed with treatment. Participants saw the sock as a tool that might improve care by providing objective, real-time information for feedback and reassurance to the wearer or health care provider. In this way, it could be used to raise awareness of safety as well as risk. At home, it could help with choosing new footwear or checking that they have effectively resolved a previous alert, and similarly, in clinical practice, it could be potentially useful when prescribing custom footwear or other offloading devices:

For me, I think it would be useful as an early warning and actually checking is my [clinical offloading] device doing what I think it’s doing.

Podiatrist 4

#### Effective Engagement

##### Challenges Accepting and Actioning Information

While the idea of a smart sensing sock was generally accessible and acceptable to participants, when questioned further about how they would use the sock, more practical questions arose, particularly about how to respond to the alert, what to look for on the affected foot, and how to find and correct the cause of the shear strain:

What can you do? You’re getting this information that’s telling you there is rubbing taking place, and is likely to cause you a problem. So, guidance or suggestions is what has to come.

P20; carer

This reaction was fueled by limited understanding of foot ulcers, associated risk factors, or what could be done to prevent them. Even when there was adequate understanding, many participants faced multiple competing demands of family, community, or employment responsibilities and reflected on how this deprioritized their self-care:

It’s difficult to prioritise yourself when you’ve got two children, you’re working, you’re trying to keep all the balls in the air. I don’t think I prioritised my health enough.

P7

Sometimes, this competition for attention was exacerbated by the sheer amount of information that needed to be absorbed after their diabetes diagnosis. The seriousness of diabetic foot ulcers and their own risk of developing them might only have come to light at the time of a foot emergency, resulting in a steep learning curve and information overload:

It was a period in our lives where I’d got so much information. Trying to compartmentalise it all.

P20; carer

Participants noted that information about foot ulcers, and especially associated risk of amputation and threat to life, could be frightening. While some participants actively sought information and felt that it reinforced the importance and practice of self-care, others appeared to be more vulnerable to the information and preferred not to know:

...don’t read up on it because it’ll scare you to death.

P4

These participants recalled the loss of close family members because of foot problems or reflected on the fact that it was information that they could not identify with, assuming that it was something that happened to other people and would not affect them. Whether it was trauma, naivety, bravado, or turning a blind eye, the reality of their own susceptibility was difficult for them to accept:

It was the worst time of my life. It took me 18 months to go to hospital to get it done in the first place. I was an ex-footballer. I was a man who was proud, if you know what I mean. I shouldn’t be losing my toe, even though what had happened. I just couldn’t get it in my head.

P17

##### Simple, Specific, and Supportive Guides

Given the importance of underestimation of risk, lack of information, and social and emotional distractions to carrying out instructions, podiatrists recommended a clear and simple decision-making tool to accompany the device. They suggested step-by-step prompts to guide the patient to safely respond to an alert; assess damage; and, critically, know when to contact their foot health team:

It sounds like you’re spoon feeding them, but sometimes it ends up being the case that you have to do that to prevent this...The time between a problem arising and how long something is done about it, within hours, diabetic feet can deteriorate, you can get a foot attack. So if that prompt is there like, “you need to check it right now” that would be really useful.

Podiatrist 4

In addition, lack of sensory information should also be addressed and supported. Both interview and focus group participants called for information in the feedback system to indicate the location of the shear strain as well as instructions on how to respond to rubbing in different areas:

You have to put yourself in their shoes. They don’t actually feel, so if you or I were to get a bit of rubbing, we’d stop what we’re doing and alternate our foot, or fix our shoe, tie our lace, because they can’t feel they haven’t a clue.

Podiatrist 3

#### Sustained Use

##### Difficulties Coping

While some were comfortable with monitoring their own health and reassured by taking measurements or recording data, others preferred to wait until clinic appointments, feeling that constant management created more, not less, anxiety. One participant who was skeptical about using the sock referred to health-monitoring devices as “worry-meters” (P5). This was a concern for the podiatrist group as well, who worried that challenges with patient engagement could be due to being overwhelmed and were hesitant to add more burden:

You just know there’ll be patients that probably wouldn’t want to have another thing to check—got to check the blood sugars, insulin like everything else. This is just another tool, but it’s another thing to do as well, and sometimes people get kind of overwhelmed.

Podiatrist 1

As we can see from the previous subthemes, participants could start their diabetic neuropathy journey without awareness, acceptance, or understanding of their foot health risk. When they experienced foot ulcers, they were understandably unprepared, challenging their ability to cope. Narratives ranged from hopelessness, including misusing their insulin in attempts to die, to emphasizing their luck in life and downplaying the misfortune of their experiences. While the fortunate few who were happy with their medical care, confident in their own abilities to self-manage their condition, and supported by family felt that their symptoms did not dominate their lives, other participants felt that they had less control:

...it’s [my foot health] totally entwined with the diabetes that really controls me, controls my feet, my eyes, all the other diabetic symptoms.

P3

Diabetic foot ulcers can escalate rapidly, and participants reported that the progression of their wounds was shocking. One participant did not even know he had diabetes until 5 days after he noticed a “small sore,” when he was admitted to hospital for emergency amputation:

I was whisked up to some theatre or other, fully conscious—because I’d eaten. I couldn’t have an epidural, so they put a needle down my leg. I was lying there, conscious—compos mentis. There was a screen up, so I couldn’t see what he was doing, but I could hear it. He took four toes off, and a little bit of the foot. I signed up to the knee, because they keep going until they run out of the bad.

P12

Where there was pain associated with the ulcer and more obvious threat to life, amputation appeared easier to understand and accept; there could even be a sense of relief after treatment. Conversely, where neuropathy masked any pain, it was more difficult to perceive the severity of the wound, and consequently, amputation could be harder to cope with. Participants described having part of their body taken away with a sense of loss and grief:

The first one I was in pain and I wanted to get rid of it. The second one, I was in no pain, and it was unexpected. It’s like someone dropping down dead; or someone dying slowly of cancer or something. That’s the difference. That one was painful, and I wanted to get rid of it. I know it was for the better. That one, I was in no pain, and it was unexpected.

P1

Participants reported lasting emotional impacts of ulceration. This could be paranoia or hypervigilance, checking their feet multiple times a day. There could be feelings of guilt or regret for not taking better care beforehand. Where there was deformity or amputation, some participants noted shame in the appearance of their feet or in being classified as disabled. One of the hardest things to deal with for participants was a lack of independence:

I’m aware people make concessions for me...and psychologically that’s horrible...I don’t like it. I don’t like being needy really.

P16

Participants reported doing what they could to manage their foot health based on their understanding and acceptance of risk factors and preventative measures. Even then, some still experienced repeated wounds and infections, often from what they considered an innocent cause, such as a small cut, a new shoe, or getting sand in between their toes on holiday. For some, there was a feeling of frustration that, whatever they tried, they could not stop it happening:

You get to the end of your tether and you think, “what? what? what can I do?”

P4

##### Gaining Control Through an Early Warning System

When speaking to participants, concerns about calibration and sensitivity were undermined by the positive possibilities of the sock. For those who recognized the risk of shear strain for themselves, if the sock was easy to use and provided reliable information, they felt that it would be more of a support than a burden. One participant said that it could be “another best friend” (P6) in the same way that she described other valued tools in her life, such as her mobile phone and well-fitted walking shoes.

Participants who reported using health devices such as continuous glucose monitors were already used to responding to alerts and appreciated the real-time feedback and prompt to take corrective action in the moment. They felt that the devices gave them more control over their health and related the sock to this same concept:

I guess I’m used to sort of reacting to information that I’ve received on, on the sort of shape of things during the course of the day. So this would just be another thing.

P16

One participant referred to the idea of an early warning system as providing “a level playing field” (P23) by compensating for lost sensation. Others felt that it could help in social situations, empowering them to speak up for themselves and take the breaks they needed rather than pushing on to keep up with others:

Especially being on your feet all day and you get busy, you get distracted. They would be great because then it would give me a bit of an alarm, so to speak, to say something’s not right, and then I need to sit out.

P4

If these benefits outweighed the burden of using the sock as well as the burden of not using it, then it would help patients manage their foot health more easily:

Well, I think it’s a good positive idea, but I don’t think it’s a game changer for diabetes. I think it’s a useful addition, like fingerprinting is a useful addition. It doesn’t make me better. It doesn’t change my life. It just helps me manage the situation better...if they were available and they work and I’m not sending them off for dry cleaning every day or, you know, that sort of thing, if the process was hard in living terms, then that would put you off. I’m sorry to give you the extra problem, but they need to fit into an ordinary sort of life, you know.

P16

## Discussion

### Summary and Comparison With Other Work

This is the first qualitative study to explore patient and podiatrist perceptions of a smart sensing device to measure shear strain for the prevention of diabetic foot ulcers. The findings suggest that potential users welcome the idea of such a device but that the experience of living with diabetic neuropathy presents several barriers to uptake and sustained effective engagement, namely, limited awareness of risk among patients and family caregivers, psychosocial challenges accepting health information and actioning health behaviors, and the emotional burdens of living with diabetic neuropathy. These barriers suggest that, for the device to be effective in improving health outcomes for this population, it should be implemented alongside a behavioral intervention.

There is limited research in this area, and our findings confirm those of the few other qualitative studies looking at patient experience of diabetic foot ulcers [33], treatment burden in long-term conditions [34], patient and podiatrist perspectives of other smart sensing wearable devices for diabetic foot ulcers [35-37], and behavioral understandings of the impacts of emotional burden on self-care behaviors [38,39]. A key novel finding of this study was that, unlike plantar pressure, which is often caused by inactivity (eg, the foot being in a single loading position for an extended period), participants considered alerts for shear strain to be associated with a different cause (ie, from a certain activity or incorrectly fitting footwear) and, consequently, that alerts would signal the need to assess and address the cause rather than simply to offload. It was not always obvious to patients how to appropriately respond to an alert for shear strain, and therefore, any future device would need to clarify the responses required. Research into smart sensing wearables for plantar pressure has found that a minimum number of alerts (1 every 2 hours) is required for optimum response [40], whereas this study suggests that, for shear strain, if the alerts are perceived as too frequent and there is no clear resolvable issue in the footwear or visible indication of rubbing on the foot (eg, redness), there is a risk that participants will assume the device to be faulty.

In addition to identifying barriers to uptake of and engagement with a smart sensing device, the findings also present potential solutions to these barriers through participant-identified adaptations to the device and its implementation. These highlight novel patient and podiatrist priorities and include using the sock to collect evidence to support clinical messaging and patient understanding of shear strain and ulceration, providing a simple decision-making tool to guide safe self-care and response to alerts, and supporting the normalization of health-monitoring behaviors to increase self-efficacy and self-advocacy regarding foot health. To further these learnings, we curated a set of guiding principles [27] derived from the outcomes of this study to support the future development of smart sensing devices for diabetic foot ulcers (Multimedia Appendix 4 [6,8,16,35-55]). These guiding principles draw on data-driven findings supported by evidence from the wider literature on this patient population and similar devices to identify key intervention features to address identified psychosocial barriers to uptake and engagement. This provision of principles addresses an urgent need to provide behaviorally informed guidance to this emerging field of smart sensing technology for diabetic foot ulcers [24]. These findings may apply to other devices that measure shear strain and be relevant to smart sensing devices for diabetic foot health more generally, and it is hoped that publishing these principles will help guide further optimization of diabetic foot health devices and the implementation of devices into standard care.

### Strengths and Limitations

The impacts of social determinants of health on individuals with diabetic neuropathy are acknowledged but not well understood [56,57] and should be considered from the outset of the research process to maximize inclusivity [58]. The strengths of this study include that people with diabetes were involved in all stages of the study, patient and podiatrist participants were purposively sampled to ensure heterogeneity of perspectives (good representation was achieved in terms of gender identity, race, age, professional experience, and patient risk factors), data collection explored feedback on the technology in the context of lived experience of diabetic foot health, and the analysis was led by a multidisciplinary team of researchers. This approach, using multidisciplinary co-design for device development and implementation and acknowledgment of contextual influences, is critical to facilitate a device to function as a clinically integrated self-care tool for prevention of diabetic foot ulcers [55]. Future research can build on the findings and guiding principles presented in this study to develop a prototype for the device and wider intervention, including supportive materials for patients, carers, and health care professionals. These supportive materials can be tested, iterated, and optimized alongside the development of the device itself. It is critical that this process continues with a focus on diversity and inclusion.

Future research can also learn from the limitations of this study. As is typical of qualitative research, participants were self-selected and, therefore, represent a portion of the population who, by their interest in taking part in research, may be more engaged in health care than those who did not respond to the invitation. Several of these patients did reflect on the fact that they had not always been so engaged and, thus, provided insights into issues that might otherwise not have been included. Participants recruited through NHS clinics were prescreened as being at high risk of diabetic foot ulcers, whereas another recruitment stream used could only prescreen by diagnosis of diabetes. All interested participants were further screened by a nonclinical research member using questions guided by author IY, who is a podiatrist. Therefore, inclusion in the study was ultimately based on their self-report of diabetic neuropathy, which is likely less reliable than clinical screening, but their diagnosis was confirmed through clinically informed screening and the narratives of their interviews, and using different recruitment streams actually helped achieve a broad sample of patients with a range of ulcer histories and experiences.

### Conclusions

This qualitative study explored patient and health care provider feedback on a novel smart sensing wearable technology (a sock and feedback system to detect and alert to shear strain) for the prevention of diabetic foot ulcers. The findings suggest that potential users welcome the idea of such a device but that the experience of living with diabetic neuropathy presents several barriers to uptake and sustained effective engagement, namely, limited awareness of risk among patients and family caregivers, psychosocial challenges accepting health information and actioning health behaviors, and the emotional burdens of living with diabetic neuropathy. This study also identified potential solutions to these barriers to improve device uptake, engagement, and sustained use. These include using the sock to collect evidence to support clinical messaging and patient understanding of shear strain and ulceration, providing a simple decision-making tool to guide safe self-care and response to alerts, and supporting the normalization of health-monitoring behaviors to increase self-efficacy and self-advocacy regarding foot health. These suggest that the device should be considered as a tool within a wider behavioral intervention designed to support self-management behaviors, for example, through specific framing of feedback messages and instructions to improve risk appraisal and build self-efficacy and by supporting health care professionals to introduce and use the device as part of their practice. A set of guiding principles was presented to support future research on device design that addresses the contextual barriers to successful uptake and long-term effective engagement identified in this study.

## Appendix

Multimedia Appendix 1 Semistructured interview guide.

Multimedia Appendix 2 Sock design features.

Multimedia Appendix 3 COREQ (Consolidated Criteria for Reporting Qualitative Research) checklist.

Multimedia Appendix 4 Guiding principles.

## Abbreviations

COREQ: Consolidated Criteria for Reporting Qualitative Research
NHS: National Health Service
PPIE: patient and public inclusion and engagement

## Data Availability

The datasets generated during this study are not publicly available to protect the identities of the participants but are available from the corresponding author on reasonable request.
