# Supplementary material for: Developing a Smart Sensing Sock to Prevent Diabetic Foot Ulcers: Qualitative Focus Group and Interview Study
Source: J Particip Med. 2025 Feb 14;17:e59608. doi: 10.2196/59608 (PMC11888051; doi:10.2196/59608)
Supplement: Multimedia Appendix 1 [file jopm_v17i1e59608_app1.docx]

**About the participant and their foot care:**

- **Can you tell me if foot ulcers are a concern for you?**
  - Do you do anything in your daily routine to manage or prevent foot ulcers?
  - How often do you do this?
  - How confident are you in your ability to manage the health of your feet?
- **Does anyone help you with your foot care?**
  - Who would be your go-to person for foot care questions/support?
  - How often do you contact them for help with your foot care?

**About the sock:**

- **Thinking about the sock itself, can you tell me your first impressions?**
- **Can you tell me if you can see yourself using this sock in your daily life?**
  - How often do you think you might use it?
  - In what situations do you think you might use it?
- **Can you tell me about any features that appeal to you?**
  - What is it about this feature that you like?
- **Can you tell me any specific features that might be a concern?**
  - How concerned are you about this feature?
  - Would this be a barrier to your using the sock at all?/ long-term?
- **Is there anything about the sock that you would like to change?**

**About the feedback system:**

- **Thinking about the feedback system, can you tell me your first impressions?**
- **Can you tell me if this feedback system would work for you in your daily life?**
- **Can you tell me about any features that appeal to you?**
  - What is it about this feature that you like?
- **Can you tell me any specific features that might be a concern?**
  - How concerned are you about this feature?
  - Would this be a barrier to your using the feedback system at all?/ long-term?
- **Is there anything about the feedback system that you would like to change?**

**General prompt examples**

- **How does/did that make you feel?**
- **Can you tell me a little bit more about that?**
- **That’s really interesting – please can you explain a bit more?**
- **That sounds really difficult – do you feel able to tell me a bit more about that?**
