# Supplementary material for: Developing a Smart Sensing Sock to Prevent Diabetic Foot Ulcers: Qualitative Focus Group and Interview Study
Source: J Particip Med. 2025 Feb 14;17:e59608. doi: 10.2196/59608 (PMC11888051; doi:10.2196/59608)

| Component | Detail | Participant and PPIE feedback | Device development |
| --- | --- | --- | --- |
| Sock |  |  |  |
|  | Tightness | Consider oedema, ease of putting on and off. Should be supportive, but not tight. Wider fit on legs as well as toe area if bandages are being worn | The sock is designed with a softer elastic that enables the user to wear them without discomfort. |
|  | Seams | Completely seamless especially at the heel and toe, but also careful that the welt stitching is not too sharp especially when stretched. | The seamless toe finish in the toe part and added pre-toe design offers further comfort along with the terry knit design |
|  | Welt | Important area for tightness consideration – wider welt area less likely to impede circulation | A double welt structure knitted with increased height knitted using a soft elastic yarn at the top of the sock gives firmness as well as holds the sock in position and prevents any tightness affecting circulation. It also helps the wearer to hold the sock firmly when wearing the sock. |
|  | Toes | Covered or padded toes are good for protection and warmth, but toeless option is good for people with burning sensation, or nail issues. Also concerns about discomfort in the toes and them needing to feel “free” rather than rubbing together | The heel and toe part of the sock have been knitted using terry knit design which offers padded or cushion effect to the wearer as well as warmth. A ‘open toe sock’ have been developed for those who would like to wear sandals and have to inspect the toes due to a wound/infection. |
|  | Fabric | Breathable/absorbing excessive moisture is an infection risk, anti-odour | The sock is made of a single jersey knit structure, which offers airflow easily through its structure. The sock is made of combed cotton fibres which could excess absorb excess moisture and nylon fibres enable the fabric to wick away the absorbed moisture to environment. |
|  | Additional | Right and left labels, grip pads – to reduce sliding | The sock is knitted with alphabets ‘L’ and ‘R’ for patients to wear the sock correctly. The heel part of the sock is designed with a Y-shaped design and terry knit, which cups/holds on to the heel and prevents the sock from sliding when donning and doffing the sock. |
| Sensors |  |  |  |
|  | Location | Ball of foot, heel, met heads, side of big toe | Prototype placement of sensors – below the heel and near the large toe |
|  | Practical | Washable, durable | The sensor components can be detached during routine wash maintenance |
| Terminal |  |  |  |
|  | Concerns | Ease of removal and reattachment, charging | At the prototype phase, this detail has not yet been addressed as the focus is on reliable sensor functioning, but considerations of associated conditions such as peripheral neuropathy and arthritis that may affect dexterity will be considered in the ergonomics of this design. |
| Feedback |  |  |  |
|  | Key elements | Haptic feedback or app were acceptable;  Indication of specific area of rubbing was preferable;  Prompts to accompany the alert to facilitate decision making (what to do, when, how urgently);  Simple clear instructions (multi-language, pictures best);  Advice about regular self-care, foot emergency, footwear fitting, when to seek medical attention. | This part of the device will be developed once the sock and sensors are shown to be functional, and their design refined to clinical standards. These comments are important design features that have been noted by participants and PPIE group that will be considered when the feedback element design begins. |

Four different sock samples were produced at Manchester Fashion Institute, Manchester Metropolitan University using a Santoni sock knitting machine after carefully reviewing a wide range of commercially available diabetic socks. The design of the sock was also based on the discussions and initial feedback from the patient groups. During the design stage, various factors, such as the thickness of the socks, ease of wear, ability to stretch easily, and seamless toe, were considered. Socks were composed of recycled Nylon, combed cotton, lycra and elastic. All the socks have a double welt at the top of the sock and a seamless toe design that offers comfort in the toe region. Except for design number 3, all the remaining socks have terry heel and toe, which provide softness and added comfort to the heel and toes. Socks with three zones allow the sock to be designed with additional lycra. This feature lets the sock stay on the leg and feet without gathering or sliding when donning and doffing.


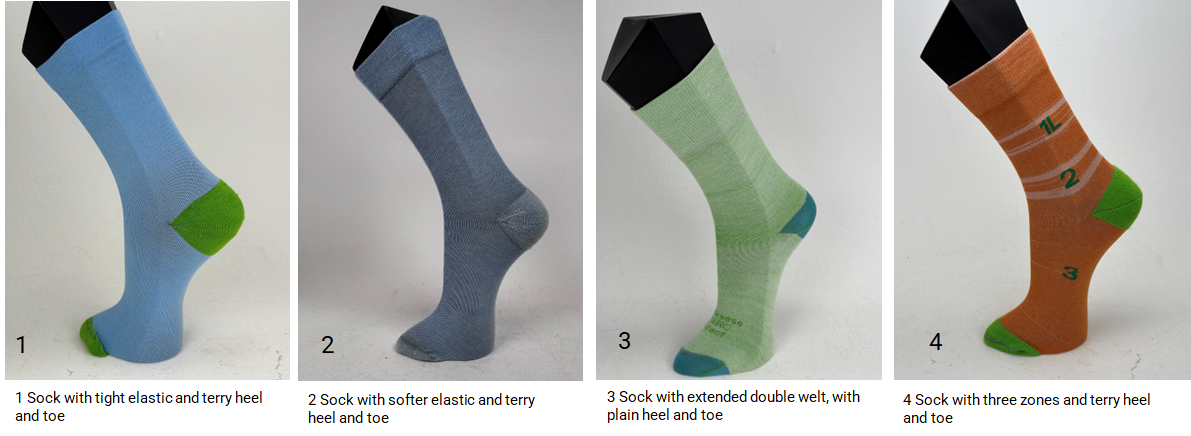

Supplement: Multimedia Appendix 2 [file jopm_v17i1e59608_app2.docx]
