# Supplementary material for: Developing a Smart Sensing Sock to Prevent Diabetic Foot Ulcers: Qualitative Focus Group and Interview Study
Source: J Particip Med. 2025 Feb 14;17:e59608. doi: 10.2196/59608 (PMC11888051; doi:10.2196/59608)
Supplement: Multimedia Appendix 3 [file jopm_v17i1e59608_app3.docx]

#### Reflexivity statement:

Reflexive thematic analysis explicitly recognises researcher input into the analysis. As such, it is important to declare researcher positioning. Author JC collected the data, conducted the analysis and write-up. She is an experienced qualitative researcher with a background in health psychology and approached the data from a constructivist standpoint [1]. She does not have lived experience of diabetes and had no previous relationship with any of the participants. Supervising the study and providing input to the analysis, was KB, an experienced qualitative researcher, Health Psychologist and expert in the Person-Based Approach with over 15 years in the field of health intervention design; IY, a Podiatrist specialising in diabetic foot care and clinical academic; and the engineering team including experts in digital diabetic foot care interventions development. To ensure credibility, all stages of the research were reviewed with the PPIE group for feedback.

|  | | Item | Guide questions/description | Manuscript section where information can be found |
| --- | --- | --- | --- | --- |
| **Domain 1: Research team and reflexivity** | | | | |
| Personal Characteristics | 1 | Interviewer/facilitator | Which author/s conducted the interview or focus group? | Method- data collection |
|  | 2 | Credentials | What was their occupation at the time of the study? | Method- data collection  Supplementary material - Reflexivity |
|  | 3 | Occupation | What was their occupation at the time of the study? | Method- data collection Supplementary material - Reflexivity |
|  | 4 | Gender | Was the researcher male or female? | Supplementary material - Reflexivity |
|  | 5 | Experience and training | What experience or training did the researcher have? | Supplementary material - Reflexivity |
| Relationship with participants | 6 | Relationship established | Was a relationship established prior to study commencement? | Method – participants Supplementary material - Reflexivity |
|  | 7 | Participant knowledge of the interviewer | What did the participants know about the researcher? e*.g. personal goals, reasons for doing the research* | Method – data collection |
|  | 8 | Interviewer characteristics | What characteristics were reported about the interviewer/facilitator? e.g. *Bias, assumptions, reasons and interests in the research topic* | Supplementary material - Reflexivity |
| **Domain 2: study design** | | | | |
| Theoretical framework | 9 | Methodological orientation and Theory | What methodological orientation was stated to underpin the study? *e.g. grounded theory, discourse analysis, ethnography, phenomenology, content analysis* | Introduction Method – data analysis |
| Participant selection | 10 | Sampling | How were participants selected? *e.g. purposive, convenience, consecutive, snowball* | Method- participants |
|  | 11 | Method of approach | How were participants approached? e*.g. face-to-face, telephone, mail, email* | Method- participants |
|  | 12 | Sample size | How many participants were in the study? | Findings- participants |
|  | 13 | Non-participation | How many people refused to participate or dropped out? Reasons? | Our recruitment method does not allow us to know why participants did not respond to our invitation to participate. |
|  | 14. | Setting of data collection | Where was the data collected? e*.g. home, clinic, workplace* | Method- data collection |
|  | 15. | Presence of non-participants | Was anyone else present besides the participants and researchers? | Method- data collection |
|  | 16. | Description of sample | What are the important characteristics of the sample? *e.g. demographic data, date* | Method - participants  Table 1 |
| Data collection | 17. | Interview guide | Were questions, prompts, guides provided by the authors? Was it pilot tested? | Method- data collection |
|  | 18. | Repeat interviews | Were repeat interviews carried out? If yes, how many? | Method – data collection |
|  | 19. | Audio/visual recording | Did the research use audio or visual recording to collect the data? | Method- data collection |
|  | 20 | Field notes | Were field notes made during and/or after the interview or focus group? | Methods- data collection |
|  | 21. | Duration | What was the duration of the interviews or focus group? | Methods- data collection |
|  | 22. | Data saturation | Was data saturation discussed? | The authors are very cautious about claims of data saturation in thematic analysis (see, for example, arguments in Braun and Clarke [2]) Data saturation for the current analysis was not aimed for, instead, authors prefer the concept of information power [3] and n>20 was considered a good sample size to aim for based on our experience in previous projects. |
|  | 23. | Transcripts returned | Were transcripts returned to participants for comment and/or correction? | n/a  (member checks with participants were not conducted, professional transcribers transcribed the interviews and researchers checked for accuracy, and the PPIE group was consulted regularly for credibility checking) |
| **Domain 3: analysis and findings** | | | | |
| Data analysis | 24. | Number of data coders | How many data coders coded the data? | Method- data analysis |
|  | 25. | Description of the coding tree | Did authors provide a description of the coding tree? | Method- data analysis.  A coding tree was not used and analysis followed reflexive thematic analysis approach and discussion with peers. |
|  | 26. | Derivation of themes | Were themes identified in advance or derived from the data? | Method- data analysis |
|  | 27. | Software | What software, if applicable, was used to manage the data? | Method- data analysis |
|  | 28. | Participant checking | Did participants provide feedback on the findings? | n/a  (participants did not provide feedback, but ongoing analysis and reflections were discussed with the PPIE group (of people living with diabetes, at risk of diabetic foot ulcers) throughout the study period as stated in Method – study design section) |
| Reporting | 29. | Quotations presented | Were participant quotations presented to illustrate the themes / findings? Was each quotation identified? e*.g. participant number* | Results |
|  | 30. | Data and findings consistent | Was there consistency between the data presented and the findings? | Results |
|  | 31. | Clarity of major themes | Were major themes clearly presented in the findings? | Results  Discussion |
|  | 32. | Clarity of minor themes | Is there a description of diverse cases or discussion of minor themes? | Results |

References:

1. Appleton J V, and King L. Journeying from the philosophical contemplation of constructivism to the methodological pragmatics of health services research. Journal of Advanced Nursing; 2002. 40(6), 641–648. PMID: 12473042

2. Braun V, and Clarke V. To saturate or not to saturate? Questioning data saturation as a useful concept for thematic analysis and sample-size rationales. Qualitative Research. Sport, Exercise and Health; 2021. 13(2): p. 201-216. DOI: 10.1080/2159676X.2019.1704846

3. Malterud K, Siersma VD, and Guassora AD. Sample size in qualitative interview studies: guided by information power. Qualitative Health Research, 2016. 26(13): p. 1753-1760. PMID: 2661397
