# Supplementary material for: Developing a Smart Sensing Sock to Prevent Diabetic Foot Ulcers: Qualitative Focus Group and Interview Study
Source: J Particip Med. 2025 Feb 14;17:e59608. doi: 10.2196/59608 (PMC11888051; doi:10.2196/59608)
Supplement: Multimedia Appendix 4 [file jopm_v17i1e59608_app4.docx]

## Multimedia appendix 4: Guiding Principles

| **User Context** | **Key design objective** | **Intervention design features** |
| --- | --- | --- |
| **Risk appraisal:** | | |
| From the data:   - Many participants had difficulty accepting their diagnosis of neuropathy which reduced their motivation to perform self-care. It appeared to be contrary to their personal experience as they felt they did have at least some feeling in their feet. In contrast, those who accepted their diagnosis were more motivated to perform self-care. - Some participants did not accept that shear pressure could be causing their ulcers. Participants who were aware that shear pressure could cause issues were more careful with their footwear. - Participants believed that shear pressure must have an external cause such as a rolled sock and were unaware of issues with changes in gait from neuropathy. - Almost half of the participants underestimated their risk of ulceration. - Podiatry participants thought the real-time feedback could help their patients become more aware of footwear or activities that are causing risky levels of shear strain and may help to reinforce their clinical messaging.   From the literature:   - People at high risk of diabetic foot ulcer have changes in gait and exhibit greater levels of shear strain than their lower-risk counterparts [1], so their exposure to risk is higher, but patients with lower acceptance of their illness show less self-care behaviour and poorer foot health [2, 3]. - Neuropathy-related illness beliefs are little understood, but one study has shown them to directly influence foot care behaviour and foot health outcome [4]. - International Working Group on the Diabetic Foot guidelines highlight the need for continuous assessment of risk and need for frequent screening [5]. | - Highlight personal risks of diabetic foot disease and how this is associated with neuropathy and shear pressure. - Personalise health information to specifically target beliefs about personal risk factors [6, 7]. - Reinforce self-efficacy to reduce emotional burden as well as risk of (re)ulceration [8, 9]. | - Provide an informational resource illustrating diabetic neuropathy and the associated risk of ulcer in simple lay language. This should include information about gait changes as well as information about variations in neuropathy (e.g. fine touch versus pressure) to address potential issues of conflict between personal experience and clinical messaging leading to confusion in perception and acceptance of the diagnosis; as well as frequently asked questions and signposting to further information where relevant. - Provide an informational resource that demonstrates causes of shear pressure in feet including potential corrective actions (e.g., safe footwear guidelines, care when walking on an incline). - Provide an informational resource that demonstrates how the sock sensors can compensate for neuropathy and help prevent foot ulcers. - Illustrate different uses of the sock to facilitate evidence collection to improve risk acceptance for patient buy-in and support ongoing effective engagement.   E.g. 1: instructions for podiatrists to use the sock as an educational tool to demonstrate that the sock can detect and alert to shear in a safe setting (in clinic).  E.g. 2: instructions for patients/carers/podiatrists on how to use the sock to evaluate new footwear/offloading device, or for reassurance that an issue has been solved.  E.g. 3: provide an additional resource that can support the sock user to record key information about when the alert occurred (checkbox: shoes, activity, time of day) for personal record, and facility to share with podiatrists. |
| **Health literacy and behaviour change:** | | |
| From the data:   - None of the participants had a clear and comprehensive understanding of what diabetic foot ulcers are, how they are caused, or how to prevent them. This was influenced by cognitive burden (information overload), and emotional overwhelm (difficulty absorbing information). - Better understanding of foot health was associated with more informed preventative behaviours - Participants who reported better understanding as well as good relationships with healthcare providers, and family support appeared more confident in their self-management - Podiatrists reported attempts to educate their patients in clinic about self-care practices but struggled to motivate them to carry them out at home. - Some participants did understand about ulcers and self-care but reported challenges performing health behaviours e.g., competing social demands, conflicts with identity.   From the literature:   - Lack of understanding about the connections between diabetes and foot ulceration is critical to address to improve both preventative and treatment-seeking behaviours [10-15]. - It is well established that in addition to competence, other factors such as opportunity and motivation are critical to behaviour change, so the provision of information alone is inadequate [16, 17]. - Patient education needs to include support that is in line with their individual lifestyle, needs and values [10, 18-20]. - An alert-based insole system for plantar pressure included offloading advice and was shown to improve adherence to alerts and modify behaviour (pre-emptively offloading) [21]. - 4-week feasibility study of a podiatrist-led health coaching intervention to facilitate smart shoe insole adoption showed improved adherence during the intervention, [22] but declined afterwards [23]. - International Working Group on the Diabetic Foot guidelines highlight the impact of health literacy and adherence to recommendations calling for improvements in patient education [5]. | - Support users to improve general foot health awareness and confidence in their own self-management - Support users to respond safely to an alert - Support healthcare providers to use the device as an educational tool for collaborative engagement with patients towards ongoing improvements to long-term self-management of foot health | - Provide an informational resource to illustrate what ulcers are including what patients should be aware of (e.g., changes in skin quality, or sensation, loss of hair on the legs) - Provide a tool to clarify self-care behaviours and best practice advice for patient self-management of foot health (e.g., how to perform daily foot checks) and support habit formation and positive feedback (e.g., self-monitoring of behaviour, buddy systems) - Provide a decision-making tool that guides a user through steps to respond to an alert, including when to seek medical attention - Pilot test all educational resources on diverse groups of people to ensure that the messaging is as inclusive and acceptable as possible, and iterate to address any barriers to understanding and acceptance - Device development needs to continue into implementation phases to allow for further iterations to support normalisation of use in clinical practice |
| **Health inequalities:** | | |
| From the data:   - Podiatrists identified that their patients were either pro-active and engaged with their health or reactive and less engaged with their health. They assumed that the device would only be acceptable or used properly by those already engaged (possibly based on demographic factors) - Our stakeholder groups (including PPIE) were engaged from the planning phase of the project and acted as inequality champions actively scrutinising research processes for potential discrimination - Knowledge and experience of ulceration was often associated with trauma, for example emergency amputation, stigma, family bereavement. High fear and low efficacy were associated with frustration and hopelessness. - Digital literacy varied across participants   From the literature:   - Multidisciplinary co-design is essential for device development and implementation to facilitate the device to function as a clinically integrated self-care tool for prevention of diabetic foot ulcer [24]. - International Working Group on the Diabetic Foot guidelines highlight interpersonal differences in foot health progression and outcomes and highlight the need for personalised care [5]. | - Support healthcare providers to facilitate behaviour change in their most vulnerable (unengaged) patients - Ensure that all universal elements of the intervention are targeted towards disadvantaged or unengaged groups. | - Provide an informational resource for podiatrists to highlight common barriers to adherence and provide strategies to improve behaviour change - Co-design intervention from the outset with diverse stakeholders to ensure good representation and mitigate against inequalities. E.g.: ensure that all supplementary education material is simple (grade 6 reading level), inclusive (available in multiple languages/pictures), and strength-based (positively framed) - Allow for personalisation of settings - Provide non digital as well as digital options for resource materials to address issues of digital exclusion, and provide other accessibility options (e.g., translations to different languages, options to increase font size or text-to-speech) |
| **Managing expectations:** | | |
| From the data:   - Participants were concerned about false alarms. - Others thought that the sock would keep their feet safe   From the literature:   - performance expectancy moderates behavioural intentions of both patients and healthcare providers to adopt or use such a device [22, 23, 25]. | - To provide information about device calibration, sensitivity and robustness. - To manage expectations for the scope of the device | - Provide clear information about what the sock can and cannot do - Provide clear instructions on washability and care, and quality testing. |

References:

- 1. Jones, A.D., et al., Plantar shear stress in the diabetic foot: A systematic review and meta‐analysis. Diabetic Medicine, 2022. **39**(1): p. e14661. PMID: 34324731
- 2. Tsai, M.-C., et al., Exploring the Relationship of Health Beliefs and Self-Care Behaviors Related to Diabetic Foot Ulcers of Type II Diabetes Mellitus Patients: A Cross-Sectional Study. International Journal of Environmental Research and Public Health, 2021. **18**(13): p. 7207. PMID: 34281144
- 3. Şahin, S. and D. Cingil, Evaluation of the relationship among foot wound risk, foot self-care behaviors, and illness acceptance in patients with type 2 diabetes mellitus. Primary Care Diabetes, 2020. **14**(5): p. 469-475. PMID: 32115378
- 4. Perrin, B.M., et al., Cognitive representations of peripheral neuropathy and self-reported foot-care behaviour of people at high risk of diabetes-related foot complications. Diabetic Medicine, 2014. **31**(1): p. 102-106. PMID: 23869945
- 5. Bus, S.A. and J.J. van Netten, A shift in priority in diabetic foot care and research: 75% of foot ulcers are preventable. Diabetes Metab Res Rev, 2016. **32 Suppl 1**: p. 195-200. PMID: 26452160
- 6. Ledger, L.J., et al., Patient perceptions and understanding of pressure ulcer risk in the community: Empirical Research Qualitative. Journal of Advanced Nursing, 2023. **79**(9): p. 3312-3323. PMID: 36919007
- 7. Jarl, G. Too little or too much fear and avoidance of activities: should we start learning from the other side? in 8th International Symposium on the Diabetic Foot, 2019, Hague, Netherlands, May 22-25, 2019. 2019. URN: urn:nbn:se:oru:diva-77282
- 8. Beattie, A.M., R. Campbell, and K. Vedhara, ‘What ever I do it’s a lost cause.’ The emotional and behavioural experiences of individuals who are ulcer free living with the threat of developing further diabetic foot ulcers: a qualitative interview study. Health Expectations, 2014. **17**(3): p. 429-439. PMID: 22429399
- 9. Kok, G., et al., Ignoring theory and misinterpreting evidence: the false belief in fear appeals. Health Psychology Review, 2018. **12**(2): p. 111-125. PMID: 29233060
- 10. Van Netten, J.J., J. Woodburn, and S.A. Bus, The future for diabetic foot ulcer prevention: A paradigm shift from stratified healthcare towards personalized medicine. Diabetes/Metabolism Research and Reviews, 2020. **36**(S1): p. e3234. PMID: 31944530
- 11. Coffey, L., C. Mahon, and P. Gallagher, Perceptions and experiences of diabetic foot ulceration and foot care in people with diabetes: A qualitative meta-synthesis. International Wound Journal, 2019. **16**(1): p. 183-210. PMID: 30393976
- 12. Fayfman, M., et al., Barriers to diabetic foot care in a disadvantaged population: A qualitative assessment. Journal of Diabetes and its Complications, 2020. **34**(12): p. 107688. PMID: 32917487
- 13. Tan, T.-W., et al., A qualitative study of barriers to care-seeking for diabetic foot ulceration across multiple levels of the healthcare system. Journal of Foot and Ankle Research, 2022. **15**(1): p. 56. PMID: 35932076
- 14. McPherson, M., M. Carroll, and S. Stewart, Patient-perceived and practitioner-perceived barriers to accessing foot care services for people with diabetes mellitus: a systematic literature review. Journal of Foot and Ankle Research, 2022. **15**(1): p. 92. PMID: 36527060
- 15. Feinglass, J., et al., How ‘preventable’ are lower extremity amputations? A qualitative study of patient perceptions of precipitating factors. Disability and Rehabilitation, 2012. **34**(25): p. 2158-2165. PMID: 22533668
- 16. Michie, S., M.M. van Stralen, and R. West, The behaviour change wheel: a new method for characterising and designing behaviour change interventions. Implement Sci, 2011. **6**: p. 42. PMID: 21513547
- 17. Alshammari, L., et al., The effectiveness of foot care educational interventions for people living with diabetes mellitus: An umbrella review. Journal of Tissue Viability, 2023. PMID: 37369610
- 18. Malki, A., et al., Factors influencing the use of therapeutic footwear in persons with diabetes mellitus and loss of protective sensation: A focus group study. PLoS One, 2023. **18**(1): p. e0280264. PMID: 36634096
- 19. Kudlová P, Chrastina J, and Xinopulos P. A patient's non-adherence to the treatment of diabetic footulcers: longitudinal case study. Nursing Perspectives/Osetrovatelske Perspektivy, 2023. **6**(1). DOI: 10.25142/osp.2023.002
- 20. Han, K.J., S. Kim, and n. null, Toward More Persuasive Diabetes Messages: Effects of Personal Value Orientation and Freedom Threat on Psychological Reactance and Behavioral Intention. Journal of Health Communication, 2019. **24**(2): p. 95-110. PMID: 30821640
- 21. Najafi, B., et al., Smarter Sole Survival: Will Neuropathic Patients at High Risk for Ulceration Use a Smart Insole-Based Foot Protection System? Journal of Diabetes Science and Technology, 2017. **11**(4): p. 702-713. PMID: 28627227
- 22. Macdonald, E.M., et al., Podiatrist-Delivered Health Coaching to Facilitate the Use of a Smart Insole to Support Foot Health Monitoring in People with Diabetes-Related Peripheral Neuropathy. Sensors, 2021. **21**(12): p. 3984. PMID: 34207743
- 23. Macdonald, E.M., B.M. Perrin, and M.I. Kingsley, Factors influencing Australian podiatrists’ behavioural intentions to adopt a smart insole into clinical practice: a mixed methods study. Journal of Foot and Ankle Research, 2020. **13**(1): p. 1-12. PMID: 32487234
- 24. Muller, I., et al., Combining qualitative research with PPI: reflections on using the person-based approach for developing behavioural interventions. Research involvement and engagement, 2019. **5**(1): p. 1-8. PMID: 31807316
- 25. Macdonald, E.M., et al., Factors influencing behavioural intention to use a smart shoe insole in regionally based adults with diabetes: a mixed methods study. Journal of Foot and Ankle Research, 2019. **12**(1): p. 29. PMID: 31139261
